# Finger counting training enhances addition performance in kindergarteners

**DOI:** 10.1111/cdev.14146

**Published:** 2024-09-18

**Authors:** Céline Poletti, Marie Krenger, Marie Létang, Brune Hennequin, Catherine Thevenot

**Affiliations:** ^1^ Institute of Psychology University of Lausanne Lausanne Switzerland; ^2^ Lea.fr, Editions Nathan Paris France

## Abstract

Our study on 328 five‐ to six‐year‐old kindergarteners (mainly White European living in France, 152 girls) shows that children who do not count on their fingers and undergo finger counting training exhibit drastic improvement in their addition skills from pre‐test to post‐test (i.e., accuracy from 37.3% to 77.1%) compared to a passive control group (39.6% to 47.8%) (*p* < .001, $\eta_{p}^{2}$ = .15). This result was replicated on a much smaller scale (37 five‐ to six‐year‐olds, mainly White European, 22 girls) but in more controlled setup and was further replicated with an active control group (84 five‐ to six‐year‐olds, mainly White European, 37 girls). Therefore, we demonstrate here for the first time that training finger counting constitutes a highly effective method to improve kindergarteners' arithmetic performance.

In the present paper, the question often asked by teachers and practitioners about whether children should be encouraged to use their fingers to solve basic calculations is addressed. As recently pointed out by Poletti et al. (2023), preschool teachers have different views regarding finger counting. Some see it as a sign of difficulty, while others associate its use with children who have advanced numerical knowledge (see also Moeller et al., 2011 for a review of the literature about a debate on this matter among neuroscientists, researchers in psychology, and mathematics educators).

What is undeniable, however, is the strong link between fingers and numbers (e.g., Butterworth, 1999; Guedin et al., 2018; Neveu et al., 2023). This association is present early in development and can be observable when, for example, 3‐year‐old children use their fingers to communicate their age (Lüken, 2019) or when 4‐year‐old children use their fingers to solve simple subtraction problems (Björklund et al., 2019). Such strategies are not limited to childhood and even adults rely on fingers in numerical contexts, such as keeping track of a counting sequence (e.g., Lucidi & Thevenot, 2014).

Nevertheless, adults rarely use their fingers to calculate a smoall sum such as 3 + 2, and such behavior would most probably be attributed to pathological difficulties in mathematics or mental disabilities (Kaufmann et al., 2011). It remains challenging to determine the specific age at which finger use for calculation begins to indicate math difficulties. Several studies in the domain of educational and developmental psychology reveal that, somehow against intuition, kindergarteners who use their fingers to solve arithmetic problems are more efficient than children who do not use this strategy (Dupont‐Boime & Thevenot, 2018; Jordan et al., 2008; Krenger & Thevenot, 2024; Poletti et al., 2022). In fact, children who use their fingers at this age are also more cognitively efficient than those who do not (Dupont‐Boime & Thevenot, 2018). The superiority in cognitive abilities observed in finger users supports Baroody's (1987) assumption that discovering the finger counting strategy is a difficult task might require the construction and mobilization of complex mathematical concepts, such as one‐to‐one correspondence (Alibali & DiRusso, 1999) or the cardinality principle (Fayol & Seron, 2005). Using fingers to represent and manipulate numbers is, in fact, an important step toward abstraction because children who use their fingers in numerical tasks have understood that a quantity can be represented by different means (Sinclair & Pimm, 2015).

Nevertheless, this positive relation between finger counting, efficiency, and cognitive skills decreases during development and eventually reverses between the ages of 8 and 9 years (Geary et al., 2004; Poletti et al., 2022; Sauls & Beeson, 1976). Children at this age who continue to count on their fingers for simple problems such as 4 + 3 or 5 + 4 have failed to internalize their strategies or, in other words, have not succeeded in shifting from externalized procedures to mental ones, such as retrieval from memory (e.g., Ashcraft, 1987) or automatized counting (Thevenot et al., 2016). In fact, efficiently using fingers to solve arithmetic problems early during development could be one of the factors favoring the internalization of external procedures. Stated differently, this could be through repetitive use of efficient finger counting strategies that children could eventually solve the problems without their fingers (Baroody, 1987; Poletti et al., 2022). This shift toward mental strategies would be made possible by more and more abstract counting strategies. More precisely, for addition problems, children initially concretely model the two collections that must be added on their fingers. For example, for 3 + 2, they represent 3 on one hand with 3 fingers raised, 2 on the other hand with 2 fingers raised, and count all the fingers raised from 1. This so‐called “ALL” strategy is usually discovered spontaneously by children, without formal instruction (Resnick, 1983). The strategy used by children at the next developmental stage is a “COUNT‐ON” strategy where one of the operands is kept in mind (either the first one: “FIRST” strategy or the larger one: “MIN” strategy) and where the counting process starts from this operand. For 3 + 2, children could therefore keep 3 in mind and count 4 and 5 on their fingers by sequentially raising one and then two fingers. This strategy is very different from the “ALL” strategy because it no longer involves concrete modeling of the problem quantities. Instead, the “COUNT‐ON” strategy consists in keeping track of the solving process, that is of the number of steps already executed. Such a sophisticated “COUNT‐ON” strategy could scaffold purely mental strategies (Carpenter & Moser, 1982).

Therefore, not only is finger counting associated with good arithmetic performance in 5‐ to 6‐year‐old children but it could also facilitate a shift toward less cognitively demanding and faster mental strategies. Then, it would be tempting to conclude that finger counting should be taught explicitly in children who have not discovered this strategy by themselves. However, jumping to this conclusion would be premature. Indeed, young children who do not count on their fingers might not be able to do so because they have not yet mastered the required numerical concepts, such as, as mentioned above, one‐to‐one correspondence, cardinality, or a sufficient abstract notion of numbers. It might therefore be pointless to teach a procedure that is not supported by an understanding of the number concept. Moreover, or consequently, it is possible that, to be efficient and to make sense for children, finger counting strategies must be discovered by the children themselves.

To address these questions, finger counting must be taught to children who do not use this strategy, and their arithmetic skills need to be assessed before and after training. This is precisely the approach that we adopted in the present study. Our approach is unprecedented because, in previous intervention studies involving fingers, either general sensory or motor finger abilities are trained, but not finger counting per se (e.g., Bonneton‐Botté et al., 2022; Gracia‐Bafalluy & Noël, 2008; Schild et al., 2020), or when finger counting is trained, it is among other skills (e.g., Ollivier et al., 2020). In fact, to our knowledge, the only study where finger counting was trained for itself is the one by Baroody (1987). However, the author was not interested in determining whether children improve their performance in an arithmetic task but rather in how children's behaviors evolve after training. The author taught the “ALL” strategy to 14 children aged between 5 and 6½ years who did not use this strategy spontaneously. Nine of these children learned the strategy after 1, 2, or 3 demonstrations, but between 12 and 21 demonstrations were necessary for the remaining 5 children. The author examined the evolution of children's strategy at 13 time points over 8 months and observed that less than one‐third of the children had shifted to mental strategies after this period. However, Baroody acknowledged that before his conclusions can be generalized, a larger sample of children would need to be tested.

Almost 40 years after this seminal study, this is what has been done in the first experiment reported here with a sample of 328 kindergarteners. A part of these children entered the training program and were pre‐ and post‐tested on their abilities to solve simple additions. The other part of children entered a passive control group. In this latter group, children were “pre‐” and “post”‐tested without any training between the testing points. In the experimental condition and, exactly as in Baroody (1987), the “ALL” strategy was taught to children. We chose to teach this strategy because it is the one predominantly used by children when they start counting spontaneously on their fingers (e.g., Carpenter et al., 1981; Carpenter & Moser, 1982; Secada et al., 1983). Teaching a modeling strategy also seems appropriate because children can understand that numbers can be concretely and analogically represented on fingers. Stated differently, training the “ALL” strategy allows children to represent and manipulate quantities on their fingers and not simply to recite a verbal sequence as in the “COUNT‐ON” strategies (Brissiaud, 1991, 1992). It is therefore predicted that the “ALL” strategy will be used spontaneously by children who already count on their fingers before our intervention and that this strategy will be used by children who respond to the training program.

If finger counting training proves to be an efficient method for enhancing children's arithmetic abilities, a greater improvement in accuracy in the addition task presented at pre‐ and post‐tests should be observed in children trained to finger counting (i.e., experimental group) compared to those in the passive control group. This effect should be particularly true in children who did not spontaneously count on their fingers before our intervention and even more so in those who were responsive to the intervention (i.e., shifting from non‐users to users of finger counting). If the positive impact of the intervention is robust, it is expected to last over time, and therefore, a decline in performance in the trained children should not be observed between an immediate post‐test and a delayed post‐test occurring 6 weeks after the end of the intervention.

The large scale at which this first experiment was conducted was facilitated by the recruitment of 28 teachers who independently implemented the protocol in their classrooms. To ensure the replicability of results in a more controlled setting, a second experiment was conducted on a smaller scale where the first author of the present paper oversaw the testing and training. Finally, in a third experiment led by the teachers, the passive control group was replaced by an active control group. In this group, children were trained to memorize the results of the same additions as those presented during the finger training program. This adjustment aimed to eliminate the possibility that the results observed in the first and second experiments were influenced by the lack of active engagement in children from the passive control group or the possibility that the trained problems were too similar to those presented at pre‐ and post‐tests.

Despite formulating precise hypotheses, our approach remains exploratory. This is because, as previously mentioned, there is no framework that definitively predicts whether children can learn a finger counting strategy and whether this newly taught strategy can indeed improve arithmetic performance. It is also plausible that children may not adopt the taught finger strategy if they lack the necessary understanding of numerical concepts.

## EXPERIMENT 1

### Method

#### Participants

**FIGURE 1:**
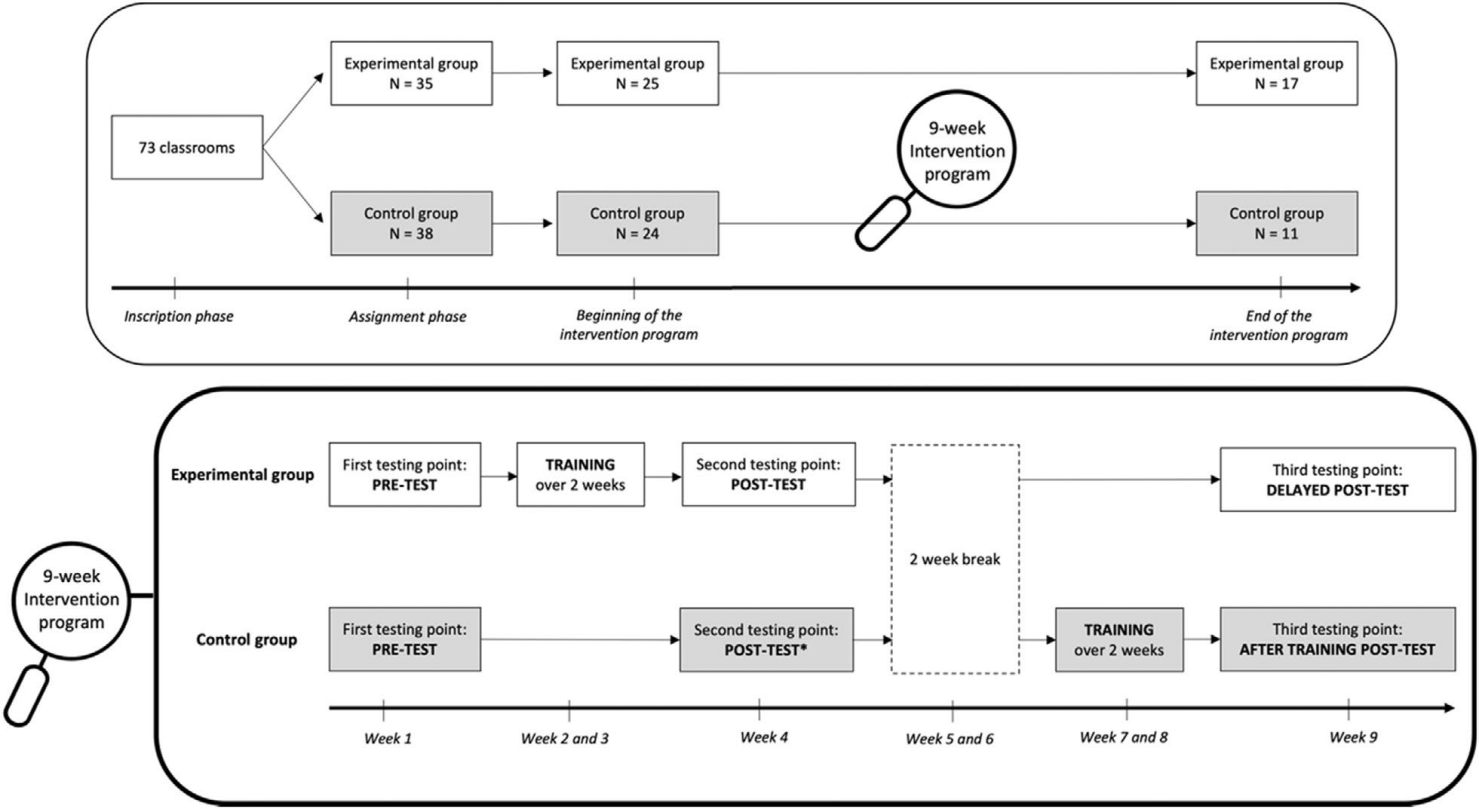
Graphical representation of the classroom repartition (top panel) and of the course of the study for the experimental and control groups (bottom panel) in Experiment 1. *Post‐test considered as pre‐test for children who entered the intervention program after being in the control group.

Kindergarteners' teachers from 73 classrooms in different parts of France signed up to participate in our experiment. At this point, the classrooms were randomly assigned to the experimental or control group. However, 24 teachers never began the experiment and 21 started but did not conduct the protocol until the end. Therefore, our final sample was constituted of 28 classrooms (17 in the experimental group, comprising 219 children and 11 in the control group, comprising 179 children) belonging to different schools and including children from various socio‐economic backgrounds. Note that the experiment took place between January and March 2022 and this high dropout rate was certainly due to the post‐COVID situation, with increased stress and workload for teachers (see Figure 1 for a graphical representation of the dropout). Moreover, among the 398 children involved in the experiment, 70 of them (28 from the experimental group and 42 from the control group) were excluded from the pool because they did not participate to the pre‐test or immediate post‐test.

Our dataset was therefore collected on a total of 328 children (191 from the experimental group and 137 from the control group) aged from 5 to 6½ years (152 girls, *M* = 69 months (5 years and 9 months), SD = 4 months, range from 60 to 77 months). More precisely, 75% of the children were aged between 5 and 6 years and the remaining 25% corresponded to children aged between 6 and 6½ years. Most of the children were White European. None of these children presented developmental disorders or disabilities. For all children, written consents were obtained from the parents before the experiment and the study was approved by the Ethics Committee of the Social and Political Science Faculty of the University of Lausanne (Decision number C_SSP_102021_00006).

In France, kindergarteners are presented at school with additions and subtractions embedded in concrete situations without formalization, either by playing, by drawing the situations, or by manipulating concrete material. There is no specific instruction concerning finger counting in the guidelines from the French Ministry of Education for kindergarteners' teachers. We were therefore confident that the children involved in our study could solve the additions presented to them and also confident that not all children would use finger counting to solve the problems.

#### Procedure

Children were recruited through their teachers who voluntarily took part in the experiment. To participate, teachers had to register on a digital pedagogical and collaborative network at www.lea.fr. This platform offers free educational resources and classroom activities and hosts several research projects about education. The platform was also used to provide teachers with all the material and procedure details necessary to implement the intervention program in their classroom. A forum where teachers could ask their questions was available throughout the study.

There were 4 successive steps for the experimental group, including a pre‐test, a training over 2 weeks, a post‐test closely following the end of the training (i.e., immediate post‐test taking place in the week following training), and a delayed post‐test (i.e., taking place 6 weeks after the end of training). For ethical concerns, these children entered the training program after they were tested for the second time (this second testing can be considered as their pre‐test) and were therefore tested a third time for what corresponded to a post‐test training for them (see Figure 1 for a graphical representation of the intervention). The experiment ended after this third testing for children in the control group, which means that there was no delayed post‐test for them. Note that for the sake of brevity, the results before and after the training program for the control group will not be presented in this paper.

##### Pre‐ and post‐test assessments

At pre‐ and post‐tests, the same 10 additions were presented to children in a face‐to‐face situation with teachers. The additions were constructed with different addends from 1 to 5 (non‐tie problems) with the second addend larger than the first one (i.e., Min + Max problems, e.g., 1 + 2, see Appendix 1 for the whole set of problems used). The sum of the problems therefore ranged from 3 to 9. Very importantly, the additions used during pre‐ and post‐tests were different from those used during training.

Each addition was written on a paper card and presented to children who were asked to solve it. More precisely, teachers asked “If there is X and then Y, how many are there in total?” (translated from the French formulation “Si il y a X et puis Y, combien cela fait‐il au total?”). When children did not give any answer or attempt to answer, another problem was presented after approximately 15 s. To avoid an uncomfortable situation for children, the time limit was reduced to 5 s after three unanswered problems. Of course, the 15‐s time limit was not applied when children were in the middle of the solving process. There was no stop criterion, which means that the 10 additions were presented systematically to all children. Additions were presented in random order but the problem 1 + 2 was always presented first to make children comfortable with an easy problem at the beginning of the task. The intervention program was designed in partnership with the fourth author of this paper, who is a kindergarten teacher hired by Nathan® Edition to assist researchers in adapting their research protocols to specific classroom's constraints and environments.

The teachers involved in the study had to fill out a data collection table with the observations they made during the tests concerning children's behaviors (see Appendix 2 for a translated version from French). They collected children's numerical responses to the addition problems, which allowed us to determine whether the problem was solved successfully (coded 1 in our analyses) or not (coded 0). This coding was used to determine accuracy in the addition task. Children's scores varying from 0 to 10 (as 10 additions were presented) were converted in percentages of correctly solved additions for our statistical analyses.

Teachers also reported whether children used their fingers (coded 1 in our analyses) or not (coded 0) to solve them. When children did not use their fingers, it was coded as a MENTAL strategy. This coding was used to create the groups of finger users versus non‐finger users. More specifically, children were considered as finger users as soon as they used their fingers at least once in the addition task. The percentages of problems solved with fingers by finger users were also calculated and included in our statistical analyses.

When children used their fingers, the teachers had to report how many hands were used and how many fingers were raised on each hand used. When children used both hands, the information collected allowed us to infer whether children represented the operands separately on each hand or across hands. For example, for 3 + 4, when each operand was represented on each hand (i.e., 3 on one hand and 4 on the other), it was coded as a “separate hand” strategy, but when the operands were represented in a continuous process across hands (i.e., 3 represented on one hand and 4 represented by raising two additional fingers on the same hand and two fingers on the other, resulting in raising 5 fingers on one hand and 2 on the other), it was coded as an “across hand” strategy. Note that the “ALL” strategy that we taught corresponds to a strategy where both hands are used with each operand represented separately on each hand. When children used one hand and represented only one operand, the information collected allowed us to determine whether it was the smaller or the larger operand that was represented. Nevertheless, as it will be described in our result section, finger users massively used their two hands to solve the problems, which is why we did not enter into such details for our analyses and descriptions when children used only one hand to count.

Finally, teachers were asked to report what children verbalized during the task for each addition solved. Our idea in collecting such protocols was to determine whether children represented the operands via a different process for sum count and addend representations (e.g., 3 + 4 is 1, 2, 3 and then 1, 2, 3, 4 and all the fingers are counted, 1, 2, 3, 4, 5, 6, 7) or via a single process (e.g., 3 + 4 is 1, 2, 3, 4, 5, 6, 7; Baroody, 1987). Unfortunately, 80% of the 2511 statements collected were too ambiguous to allow a proper categorization and this is the reason why we decided not to exploit this variable.

##### Training program

The training program consisted of six 10‐min collective teaching sessions (i.e., 3 sessions per week over 2 weeks) during which children were taught to solve addition problems using their fingers. The groups of children trained could vary between 5 and 24 children. A total of 15 additions were used during the training session. The additions were constructed with addends from 1 to 5 and sums from 3 to 10. Tie (e.g., 2 + 2) and non‐tie problems were presented, but for non‐tie problems, only problems with the first addend larger than the second (i.e., Max + Min problems) were used (e.g., 3 + 2). During each training session, 5 of those addition problems were presented to the whole classroom and solved using fingers according to teachers' instructions. The first addition problem of each session was always pre‐determined by the experimenter, and the other problems were presented in a random order by teachers. All the 15 problems were presented during the first week of the training and were repeated during the second week (see Appendix 3 for the 6 whole sets of problems used during the training session). As already stated, the training problems were different from the problem used at pre‐ and post‐tests.

**FIGURE 2:**
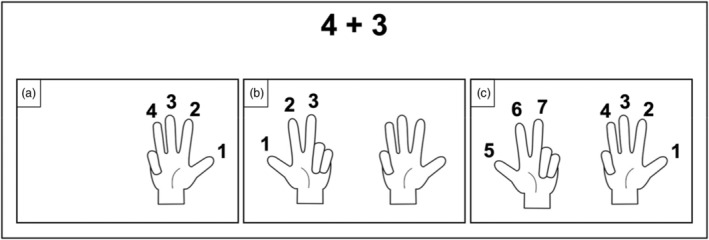
Illustration of the “ALL” strategy demonstration on fingers during the training of Experiment 1, Experiment 2, and Experiment 3. a) First operand represented; b) Second operand represented; c) All fingers counted again

Each addition was written on a paper card or directly on the board and remained in children's sight throughout the solving process. Each training session consisted of 2 steps. The first step, called “Teacher demonstration,” involved teaching children how to solve an addition problem with an “ALL” strategy on fingers. Teachers displayed the addition problem used for the demonstration (e.g., 4 + 3) on the board and asked children “If there is 4 and then 3, how many are there in total?”. Teachers began the demonstration by explaining “To do this, I'm going to count 4 on my fingers, one, two, three, four.” Teachers represented 4 on their right hand by raising each finger one by one until four (see Figure 2a). They continued the demonstration by explaining “And now, I'm going to count 3 on my fingers, one, two, three.” Teachers represented 3 on their left hand by raising each finger one by one until three (see Figure 2b). They completed the demonstration by explaining “Then, I'm going to recount all the fingers raised one by one: 1, 2, 3, 4, 5, 6, 7.” Teachers enumerated the number sequence while moving each finger sequentially when the number corresponded to the finger collection constructed was uttered (see Figure 2c). Finally, teachers concluded “So, if there is 4 and then 3, that makes 7!”. Note that the teachers' verbal statements reported here are translated from French.

The second step, called “Application of the teacher demonstration,” involved asking children to reproduce the procedure shown by the teacher using the same addition as the one used during the demonstration. During this step, teachers corrected children who had difficulty in reproducing the procedure. Following our example, teachers had therefore to reintroduce the addition problem “4 + 3” and explained: “Now that you have seen how I have solved 4 + 3, let's try to do it altogether.” Teachers drew the attention of children on the numbers written to the paper card and explained “We raise up 4 fingers, one by one, on one hand, one, two, three, four.” Teachers had to wait and check that all the children had raised up 4 fingers and then explained “We raise up 3 fingers, one by one, on the other hand, one, two, three.” Teachers had to wait and check that all the children had raised up 3 fingers on the other hand and explained “Now, we are going to count all the fingers raised up without forgetting any: 1, 2, 3, 4, 5, 6, 7.” After the collection of fingers was counted, teachers concluded “So, if there is 4 and then 3, that makes…? Yes! 7!”. Teachers always represented the first operand of the addition using their right hand and the second operand on their left hand. However, children could use the hand they wanted to represent the first operand. This second step was repeated for the remaining 4 addition problems of the session.

### Results

**FIGURE 3:**
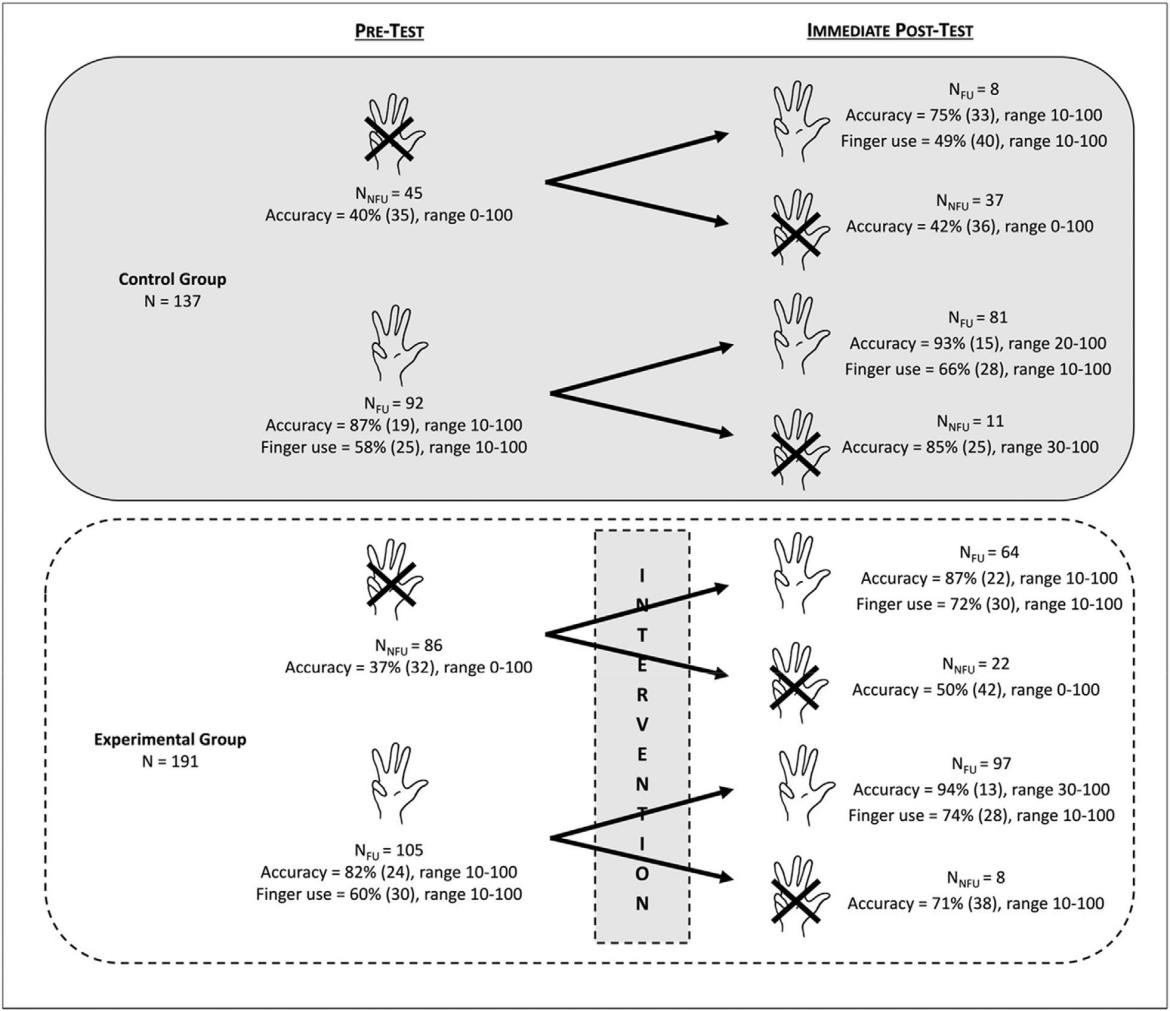
Numbers of children, percentages (with SD) and ranges of correctly solved additions, and percentages (with SD) and ranges of finger use across the different trajectories between pre‐ and immediate post‐test for the control and experimental groups in Experiment 1. FU, finger users; NFU, non‐finger users.

Before the statistical results directly related to our hypotheses are presented, a depiction of the different trajectories that children followed in their use of fingers during our training program is found in Figure 3.

#### Evolution between pre‐test and immediate post‐test for the experimental and control groups

##### Percentages of finger use in the arithmetic task

**FIGURE 4:**
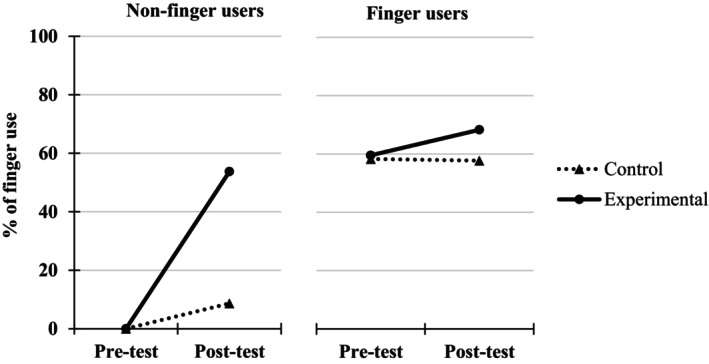
Percentages of finger use at pre‐ and immediate post‐tests in children from the experimental and control groups as a function of their behavior at pre‐test (finger users or not) in Experiment 1.

A 2 (Group: experimental, control) × 2 (Testing point: pre‐test, immediate post‐test) ANOVA, with the first factor as a between‐measure and the second one as a repeated measure, was performed on the percentages of finger use. This analysis was carried without entering the Finger use group (i.e., finger users vs. non‐finger users at pre‐test) as a variable because the percentages of finger use in the non‐finger user groups are inherently 0, which precludes inferential analyses (i.e., variance of 0 in this group and confound between the independent and the dependent variables) (see however Figure 4 for a descriptive presentation of the results obtained in finger and non‐finger users).

The analysis revealed a significant Group × Testing point interaction, *F*(1, 326) = 40.3, $\eta_{p}^{2}$ = .11, *p* < .001, showing that the increase in finger use between pre‐test and immediate post‐test was higher for the experimental group (from 32.7% at pre‐test to 61.7% at immediate post‐test, +29.0%) than for the control group (from 39.1% at pre‐test to 41.6% at immediate post‐test, +2.5%). Post‐hoc analyses further revealed that this increase in finger use was significant only for the experimental group, *t*(326) = 10.74, *p* < .001, and not for the control group, *t*(326) = 0.78, *p* = .437.

##### Percentages of correct responses in the addition task

**FIGURE 5:**
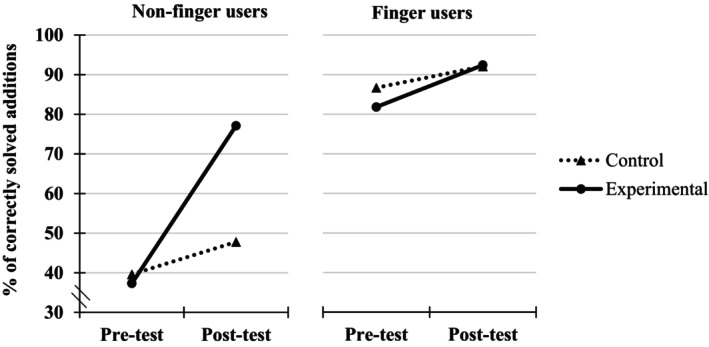
Percentages of correct responses in the addition task at pre‐ and immediate post‐tests in children from the experimental and control groups as a function of their behavior at pre‐test (finger users or not) in Experiment 1.

A 2 (Group: experimental, control) × 2 (Finger use group: finger user, non‐finger user at pre‐test) × 2 (Testing point: pre‐test, immediate post‐test) ANOVA, with the first two factors as between‐measures and the last one as a repeated measure, was performed on the percentages of correctly solved additions (Figure 5).

The analysis revealed a significant Group × Testing point interaction, *F*(1, 324) = 32.0, $\eta_{p}^{2}$ = .09, *p* < .001, showing that the improvement in the percentages of correctly solved additions was higher in the experimental group (from 59.6% at pre‐test to 84.7% at immediate post‐test, +25.1%) than in the control group (from 63.1% at pre‐test to 69.9% at immediate post‐test, +6.8%). This interaction remained significant when only non‐finger users were considered, *F*(1, 129) = 23.4, $\eta_{p}^{2}$ = .15, *p* < .001, showing again that the effect of training was higher for the experimental group (from 37.3% at pre‐test to 77.1% at immediate post‐test, +39.8%) than for the control group (from 39.6% at pre‐test to 47.8% at immediate post‐test, +8.2%) within this subpopulation.

This interaction was further modulated by Finger use group (i.e., Group × Finger use group × Testing point, *F*(1, 324) = 16.4, $\eta_{p}^{2}$ = .05, *p* < .001). Indeed, the experimental effect just described was higher in the group of 86 non‐finger users than in the group of 105 finger users. In fact, the positive effect of the training was significant only for non‐finger users (as seen above, from 37.3% at pre‐test to 77.1% at immediate post‐test, +39.8%, *t*(129) = 4.83, *d* = 0.89, *p* < .001) and not for finger users (from 81.8% at pre‐test to 92.4% at immediate post‐test, +10.6%, *t*(195) = 1.71, *d* = 0.24, *p* = .089).

Finally, analyses in the sub‐samples of non‐finger users from the experimental group who responded to our intervention (i.e., who shifted to finger use at post‐test) or not were conducted. Concerning the 74% of children (*N* = 64) who shifted to a finger counting strategy at immediate post‐test, the percentages of correctly solved additions went from 32.5% at pre‐test to 86.6% at immediate post‐test (i.e., +54.1%), *t*(63) = 12.70, *d* = 1.59, *p* < .001. In contrast, the remaining 26% of children (*N* = 22), who still did not use their fingers to solve the additions at immediate post‐test, did not show accuracy improvement (from 51.4% at pre‐test to 49.5% at immediate post‐test, −1.9%), *t*(21) = 0.59, *d* = 0.13, *p* = .559.

#### Evolution between pre‐test, immediate, and delayed post‐tests for non‐finger users (at pre‐test) from the experimental group

**FIGURE 6:**
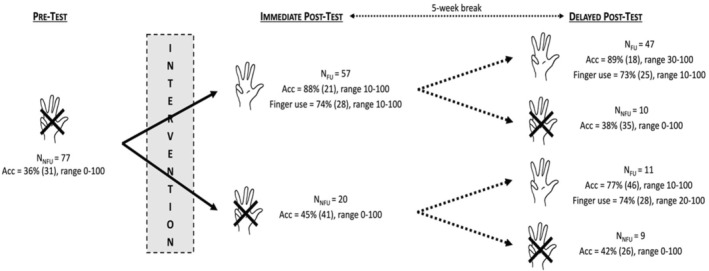
Numbers of non‐finger users, percentages (with SD) and ranges of correctly solved additions, and percentages (with SD) and ranges of finger use across the different trajectories between pre‐test, immediate post‐test, and delayed post‐test for the experimental group in Experiment 1.

As explained in the Method section, children from the experimental group took a delayed post‐test 6 weeks after the training. Out of the 191 children from the original sample, 23 did not take the delayed post‐test, resulting in a sample of 168 children. The following analyses will focus on the 77 non‐finger users at pre‐test, who constitute the critical sample for our rational (see Figure 6).

##### Percentages of finger use in the arithmetic task

As attested by the result of a t‐test, the percentages of finger use did not differ significantly between immediate (54.5%) and delayed (50.4%) post‐tests, *t*(76) = 0.95, *d* = 0.11, *p* = 348. Moreover, as represented in Figure 6, among the 57 children who became finger‐users at the immediate post‐test, 82% (*N* = 47) continued using their fingers to solve the additions at delayed post‐test, which is significantly higher than the 18% (*N* = 10), who abandoned the finger counting strategy, *p* < .001.

##### Percentages of correct responses in the addition task

A one‐way ANOVA, with the factor Testing point (pre‐test, immediate post‐test, and delayed post‐test) as a repeated measure, was performed on the percentages of correctly solved additions. The main effect of this variable was significant, *F*(2, 152) = 65.3, $\eta_{p}^{2}$ = .46, *p* < .001, showing that the percentages of correctly solved additions were higher at post‐tests (76.6% and 75.1%, at immediate and delayed post‐tests, respectively) than at pre‐test (36.2%). Post‐hoc analyses further revealed that performance improvement between pre‐test and immediate post‐test (+40.4%, *t*(76) = 9.02, *p* < .001) and between pre‐test and delayed post‐test (+38.8%, *t*(76) = 9.53, *p* < .001) was both significant. Moreover, there was no significant difference in performance between immediate and delayed post‐tests (−1.6%, *t*(76) = 0.45, *p* = .651). In fact, the improvement in accuracy between pre‐test and immediate post‐test did not differ significantly from the improvement between pre‐test and delayed post‐test, *t*(76) = 0.45, *p* = .651, showing that the benefit of the training had not decreased after 6 weeks.

#### Description of the types of finger strategies used by children

The percentages of strategies in which children represented the operands on two hands to solve the problems are depicted in Table 1. Additionally, the percentages of problems for which they represented the operands separately on each hand (i.e., the strategy we taught) rather than continuously across hands are also provided.

**TABLE 1:** Percentages of strategies involving two hands in the different groups of children involved in our experiment (and % of separate strategies in bracket) in Experiment 1.

Trajectory	Experimental group		Control group	
	Pre‐test	Post‐test	Pre‐test	Post‐test
NFU‐FU	—	98.0 (92.5)	—	100 (71.9)
FU‐NFU	83.3 (75.0)	—	88.6 (77.8)	—
FU‐FU	95.2 (77.0)	93.7 (86.1)	85.5 (73.1)	90.9 (70.9)

Abbreviations: FU, finger users; NFU, non‐finger users.

As obvious from Table 1 and as expected, children massively represented the operands on two hands, either spontaneously or after our intervention. Due to these minor variations in the strategies used by children, further analyses were not conducted.

### Discussion

Out of the 328 children involved in this first experiment, there were more children who spontaneously used their fingers in an addition task (*N* = 197) than children who did not use their fingers at all (*N* = 131). As it is obvious from Figure 5 and in replication to previous studies (e.g., Dupont‐Boime & Thevenot, 2018; Jordan et al., 2008), finger users at pre‐test presented a higher percentage of correctly solved additions (i.e., 84.1%) than non‐finger users (i.e., 38.1%). As predicted, the strategy massively used spontaneously by children was the “ALL” strategy (Table 1), the one we taught to children.

As already stated and supported by a higher increase in finger counting after the training program in the experimental than in the control group, we show in this first experiment that it is possible to teach the “ALL” strategy to kindergartners. In fact, almost 75% of the children who did not count on their fingers at pre‐test responded positively to our training or, in other words, used finger counting during the immediate post‐test (massively using the strategy taught). Moreover, we established here that our training program significatively enhanced children's arithmetic skills because the increase in performance between pre‐ and post‐test was higher in the experimental group than in the control group. This effect was created by non‐finger users at pre‐test and was even more pronounced when only children who responded positively to the training were considered (+54.1% between pre‐ and post‐test in the percentages of correctly solved additions).

Furthermore, the positive effects of our training observed in the experimental group persisted over time, as evidenced by the fact that 6 weeks after the end of the training, the percentage of finger use and performance of children in the experimental group had not decreased compared to the immediate post‐test.

Despite the highly encouraging results obtained in this initial experiment, it is important to note that the extensive scale at which it was conducted was facilitated by the fact that the intervention program (i.e., pre‐test, training, and post‐test) was entirely led by teachers. In other words, the experimental environment of the study was not under the full control of expert scientific researchers, whose responsibilities were limited to protocol design and providing instruction to the teachers. To address this potential limitation and ensure the replicability of the results obtained in this first experiment in a more traditional and controlled setting, the entire protocol was reconducted in Experiment 2 on a much smaller scale, led by the first author of the present paper.

## EXPERIMENT 2

### Method

#### Participants

Kindergarteners from 5 classrooms based in Switzerland were enrolled in the experiment, which took place between November and December 2023. Three classrooms, comprising 23 children, were randomly assigned to the experimental group, while the remaining two classrooms, with 22 children, were designated as the control group. However, 8 children (4 from the experimental group and 4 from the control group) were excluded from the pool because they did not participate to the pre‐ or post‐test. Therefore, our dataset was collected on a total of 37 children (22 girls) (i.e., 19 in the experimental group and 18 in the control group) aged from 5 to 6½ years (M = 69 months (5 years and 9 months), SD = 4 months, range from 63 to 78 months). More precisely, 89% of the children were aged between 5 and 6 years, while the remaining 11% corresponded to children aged between 6 and 6½ years. Most of the children were White European. None of these children presented developmental disorders or disabilities. For all children, written consents were obtained from the parents before the experiment and the study was approved by the Ethics Committee of the Social and Political Science Faculty of the University of Lausanne (Decision number C_SSP_012022_00002).

Children recruited for this second experiment were from a different country than those in the first experiment reported in this paper, but the school curricula are very similar between the two countries. Therefore, we were still confident that children in this second experiment could solve the addition problems they were presented with and that not all the children would use their fingers to solve the problems.

#### Material and procedure

The material and procedure were the same as in Experiment 1, except that the intervention (i.e., pre‐test, training, and post‐test) was led by the first author of the present paper instead of teachers and that children did not undergo a delayed post‐test.

### Results

Out of the 37 children involved in this second experiment, 16 children spontaneously counted on their fingers to solve the additions during the pre‐test (with 9 in the control group and 7 in the experimental group), while 21 children did not use this strategy (with 9 in the control group and 12 in the experimental group).

#### Evolution in performance between pre‐test and immediate post‐test for the experimental and control groups

##### Percentages of finger use in the addition task

**FIGURE 7:**
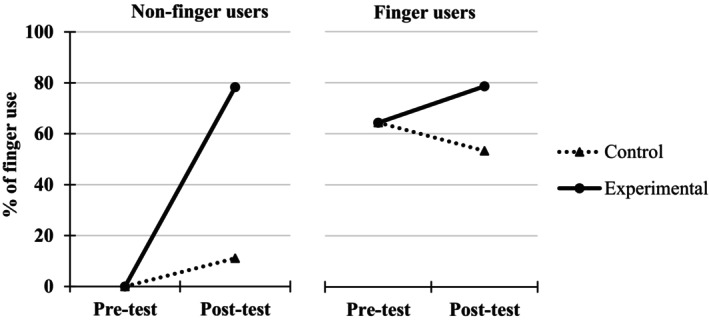
Percentages of finger use at pre‐ and post‐tests in children from the experimental and control groups as a function of their behavior at pre‐test (finger users or not) in Experiment 2.

A 2 (Group: experimental, control) × 2 (Testing point: pre‐test, post‐test) ANOVA, with the first factor as a between‐measure and the second one as a repeated measure, was performed on the percentages of finger use (Figure 7).

The analysis revealed a significant Group × Testing point interaction, *F*(1, 35) = 16.5, $\eta_{p}^{2}$ = .32, *p* < .001, showing that the increase in finger use between pre‐test and immediate post‐test was higher for the experimental group (from 23.7% at pre‐test to 78.4% at post‐test, +54.7%), whereas for the control group, there was no variation between pre‐ and post‐test (32.2% at both testing points).

##### Percentages of correct responses in the addition task

**FIGURE 8:**
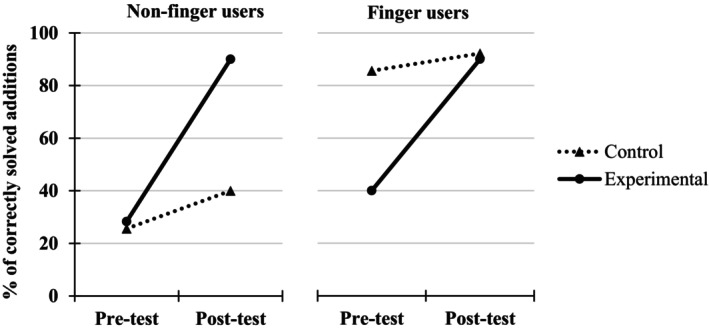
Percentages of correctly solved additions at pre‐ and post‐tests in children from the experimental and control groups as a function of their behavior at pre‐test (finger users or not) in Experiment 2.

A 2 (Group: experimental, control) × 2 (Finger use group: finger user, non‐finger user) × 2 (Testing point: pre‐test, post‐test) ANOVA, with the first two factors as between‐measures and the last one as a repeated measure, was performed on the percentages of correctly solved additions (Figure 8).

The analysis revealed a significant Group × Testing point interaction, *F*(1, 33) = 35.5, $\eta_{p}^{2}$ = .52, *p* < .001, showing that the improvement in the percentages of correctly solved additions between pre‐ and post‐test was higher in the experimental group (from 34.2% at pre‐test to 90.0% at post‐test, +55.8%) than in the control group (from 55.6% at pre‐test to 66.1% at post‐test, +10.5%). This interaction remained significant when only non‐finger users were considered, *F*(1, 19) = 19.5, $\eta_{p}^{2}$ = .51, *p* < .001, showing again that the improvement in the percentages of correctly solved additions was higher for the experimental group (from 28.3% at pre‐test to 90.0% at post‐test, +61.7%) than for the control group (from 25.6% at pre‐test to 40.0% at post‐test, +14.4%).

Furthermore, the lack of interaction between the three variables (*F* < 1, *p* = .800) suggests that the positive effect of training was not higher for non‐finger users compared to finger users.

A sub‐analysis focusing solely on non‐finger children at pre‐test who responded to the finger counting training was irrelevant here because out of the 12 non‐finger users in the experimental group, 11 switched to the finger strategy at post‐test.

### Discussion

In this second experiment, the crucial result obtained in the first experiment was replicated, indicating that a finger training program has a significantly positive impact on children's addition performance compared to the control group. The only difference between Experiment 1 and Experiment 2 was that Experiment 2 was conducted in a tightly controlled setting by the first author of the present paper, whereas Experiment 1 was led by teachers. Hence, we can assert with confidence that the outcomes of Experiment 1 were not influenced by any liberties that teachers may have taken with the provided protocol. However, the fact that this second experiment was conducted by an experimenter who was not blind to the study's hypotheses might have biased our results. Nevertheless, it must be reminded that the teachers who conducted the first experiment were blind to the hypotheses, and similar results were obtained. Thus, the lack of blindness to the hypotheses is unlikely to explain our results.

Note however that contrary to what was observed in Experiment 1, the improvement in performance in the experimental group was obtained regardless of whether children were already finger users or not at pre‐test. Nevertheless, this result cannot be considered reliable because, as depicted in Figure 8, it was due to the fact that finger users at pre‐test in the experimental group behaved atypically. Indeed, they did not present the typical superiority in performance compared to non‐finger users (e.g., Dupont‐Boime & Thevenot, 2018; Jordan et al., 2008). This is an unfortunate consequence of the small number of participants in each of the groups in this small‐scale experiment.

Nonetheless, as previously stated and despite the small number of children involved here, we once again demonstrate that arithmetic performance in non‐finger users is considerably boosted by finger counting training. Still, before definite conclusions about the benefits of finger training can be put forward, one last methodological precaution needed to be taken in a third experiment. Indeed, in Experiment 1 and Experiment 2, performance of the group undergoing experimental training was compared to that of a passive control group. The results could therefore potentially be influenced by a lack of active engagement in children from the passive control group or because the trained problems were too similar to those presented at pre‐ and post‐tests. To address this potential limitation, children in Experiment 3 were assigned to an active control group instead of a passive one. In this active control group, they were required to learn the same additions as the experimental group through rote memorization.

## EXPERIMENT 3

### Method

#### Participants

Kindergarteners from 9 classrooms in different parts of France were enrolled in the experiment, which was conducted between January and May 2023. Four classrooms were randomly assigned to the experimental group (48 children), while the remaining 5 classrooms were assigned to the active control group (56 children). However, 20 children (18 from the experimental group and 2 from the active control group) were excluded from the pool because they did not participate to the pre‐ or post‐test. Our dataset was therefore collected on a total of 84 children (37 girls) (i.e., 30 were in the experimental group and 54 were in the active control group) aged from 5½ to 6½ years (*M* = 69 months (5 years and 9 months), SD = 4 months, range from 64 to 78 months). More precisely, 74% of the children were aged between 5 and 6 years, while the remaining 26% were aged between 6 and 6½ years. Most of the children were White European. None of these children had developmental disorders or disabilities. For all children, written consents were obtained from the parents before the experiment.

#### Procedure

The procedure in the finger training group was identical to that in Experiment 1 and was once again led by teachers. However, the passive control group was replaced by an active control group. The timeline for the active control group matched that of the experimental group (and thus the passive control group in Experiment 1). More precisely, during six 10‐min collective sessions (i.e., 3 sessions per week over 2 weeks), children in the active control group were asked to learn the answers of addition problems by rote. The same 15 additions used during the training of the experimental group were presented (Appendix 3) along with their solutions. During each training session, 5 of these additions were presented one by one to the whole class with their solutions, either on a paper card or on the blackboard. After posting each problem, teachers read it twice (e.g., 4 plus 3, equals 7). The first time, teachers read the problem on their own, and the second time, they read it with the children. Teachers had to ensure that all children participated and correctly repeated the problems. In the third step, teachers hid the solution to the problem and read it again, prompting the whole class to produce the solution. Once all the problems were presented, teachers had to repeat the same procedure for each problem in the same order. Finally, teachers presented each problem once again, systematically hiding the answer so that the children could produce it.

##### Pre‐ and post‐test assessments

Children from the experimental and active control groups were asked to solve 6 addition problems. The additions were the same as those used in Experiment 1, except that problems involving 1 were not presented (i.e., 1 + 2, 1 + 3, 1 + 4, and 1 + 5). These 1 + *N* problems were excluded because it is suspected that they can be solved using a number‐after‐rule, which involved retrieving the next number after *N* in the numerical verbal sequence (e.g., Bagnoud et al., 2021; Baroody, 1995). Thus, we believe that these problems were not particularly well‐suited for rote learning. Excluding the 1 + *N* problems also allowed us to determine whether the results obtained in Experiment 1 and Experiment 2 were not due to the particular nature of these problems.

### Results

Out of the 84 children involved in this third experiment, 53 children spontaneously counted on their fingers to solve additions during the pre‐test (with 32 in the active control group and 21 in the experimental group), while 31 children did not this strategy (with 22 in the active control group and 9 in the experimental group).

#### Evolution in performance between pre‐test and immediate post‐test for the experimental and active control groups

##### Percentages of finger use in the addition task

**FIGURE 9:**
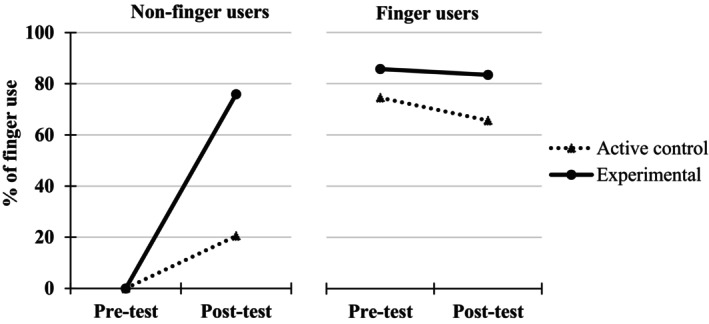
Percentages of finger use at pre‐ and post‐tests in children from the experimental and active control groups as a function of their behavior at pre‐test (finger users or not) in Experiment 3.

A 2 (Group: experimental, active control) × 2 (Testing point: pre‐test, immediate post‐test) ANOVA, with the first factor as a between‐measure and the second one as a repeated measure, was performed on the percentages of finger use (Figure 9).

The interaction between the two factors, Group × Testing point, was marginally significant, *F*(1, 82) = 3.66, $\eta_{p}^{2}$ = .04, *p* = .059, showing that the increase in finger use between pre‐ and post‐test tended to be higher for the experimental group (from 60.0% at pre‐test to 81.1% at immediate post‐test, +21.1%) than for the active control group (from 44.1% at pre‐test to 47.2% at immediate post‐test, +3.1%). Post‐hoc analyses revealed that this increase in finger use was significant only for the experimental group, *t*(82) = 2.79, *p* = .007, and not for the active control group, *t*(82) = 0.54, *p* = .590.

##### Percentages of correct responses in the addition task

**FIGURE 10:**
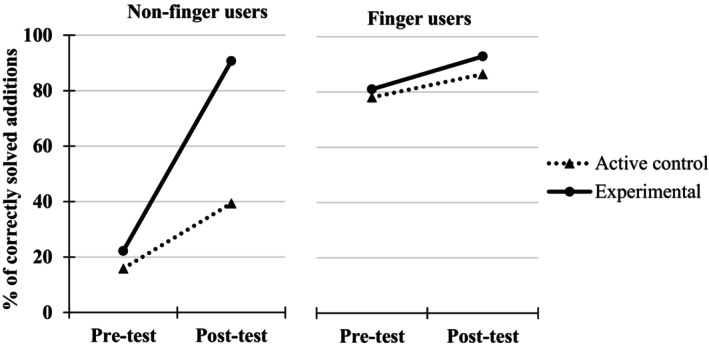
Percentages of correctly solved additions at pre‐ and post‐tests in children from the experimental and active control groups as a function of their behavior at pre‐test (finger users or not) in Experiment 3.

A 2 (Group: experimental, active control) × 2 (Finger use group: finger user, non‐finger user) × 2 (Testing point: pre‐test, immediate post‐test) ANOVA, with the first two factors as between‐measures and the last one as a repeated measure, was performed on the percentages of correctly solved additions (Figure 10).

The analysis revealed a significant Group × Testing point interaction, *F*(1, 80) = 11.19, $\eta_{p}^{2}$ = .12, *p* = .001, showing that the improvement in the percentages of correctly solved additions was higher in the experimental group (from 51.6% at pre‐test to 91.8% at immediate post‐test, +40.2%) than in the active control group (from 47.0% at pre‐test to 62.9% at immediate post‐test, +15.9%). This interaction remained significant when only non‐finger users were considered, *F*(1, 29) = 11.8, $\eta_{p}^{2}$ = .29, *p* = .002, showing again that the improvement in the percentages of correct additions solved was higher for the experimental group (from 22.2% at pre‐test to 90.7% at post‐test, +68.5%) than for the active control group (from 15.9% at pre‐test to 39.4% at post‐test, +23.5%).

This interaction was further modulated by the Finger use group (i.e., Group × Finger use group × Testing point, *F*(1, 80) = 8.13, $\eta_{p}^{2}$ = .09, *p* = .006). Indeed, the experimental effect just described was higher in the group of 31 non‐finger users than in the group of 53 finger users. In fact, the positive effect of the training was significant only for non‐finger users (as seen above, from 22.2% at pre‐test to 90.7% at post‐test, +68.5%, *t*(29) = 3.44, *d* = 1.36, *p* = .002) and not for finger users (from 81.0% at pre‐test to 92.9% at post‐test, +11.9%, *t*(51) = 0.46, *d* = 0.13, *p* = .651).

A sub‐analysis focusing solely on non‐finger users at pre‐test who responded to the finger counting training was irrelevant here because out of the 9 non‐finger users in the experimental group, 7 switched to the finger strategy at post‐test.

### Discussion

In this third experiment, the crucial result of Experiment 1 and Experiment 2 that training finger counting improves children's performance in an addition task was replicated. Unlike the previous experiments, this conclusion was reached by comparing improvement in addition performance between the experimental group and an active control group, where children had to learn a series of additions by rote memorization. The additions learned in the active control group were strictly the same as those trained in the experimental group. We can therefore conclude that the results obtained in the previous experiments were not due to a lack of engagement from children in the passive control group. Moreover, it can no longer be argued that the positive results we obtained in the previous experiments were due to the similarity between the trained additions and those used for the pre‐ and post‐tests in the experimental group. Indeed, this similarity was the same in the active control group, but the transfer of learning was lower in the active control group than in the experimental group.

It is also worth mentioning that the results of Experiment 3 were obtained while 1 + *N* problems were removed from the material, which indicates that the results in the previous experiments were not due to the particularity of these problems. As a matter of fact, we conducted a second verification of this point by re‐conducting the statistical analyses of Experiment 1 without including 1 + *N*, and the results were consistent.

## GENERAL DISCUSSION

The aim of the present study was to determine whether children who do not count on their fingers can be trained to do so and whether this training would result in enhanced performance. The results obtained in the first experiment provided a positive answer to these questions because they revealed, for the first time, that training finger counting to solve basic additions is not only possible but also constitutes a highly effective method to improve children's performance. These results were replicated in a second experiment led by a professional researcher instead of teachers and again in a third experiment where the passive control group was replaced by an active control group. Given that the results of Experiment 1 were replicated twice by taking additional methodological precautions, we think that they can be reliably discussed here.

In this first experiment and in replication to the results reported in previous literature (e.g., Dupont‐Boime & Thevenot, 2018; Jordan et al., 2008), we show that more than half of children (i.e., 60%) aged between 5 and 6 years use their fingers to solve simple addition problems. Consistent with past studies, we also find that children who count on their fingers are more accurate (84.1% of additions correctly solved) than children who do not (38.1%, see Figure 5). The original findings of this experiment reveal that children who underwent finger counting training exhibit greater improvement in an addition task between pre‐ and post‐tests (+25.1%) compared to children in the control group (+6.8%). This improvement in the experimental group was particularly notable in children who did not originally count on their fingers. Their percentages of correctly solved problems went from 37.3% to 77.1% in the addition task, compared to children who already used this strategy before the intervention (from 81.8% to 92.4% between pre‐ and post‐tests). Moreover, the positive effect of finger counting training in non‐finger users was driven by children who responded to the intervention, namely those who shifted from no finger use to finger use between pre‐ and post‐test. Indeed, while the 74% of children who responded to the training saw their success rate in the addition task increase from 32.5% to 86.6% (+54.1%), children who did not respond to the training maintained around a 50% success rate in both the pre‐ and post‐tests. This underscores that the adoption of the finger counting strategy by the majority of children in our sample was responsible for the drastic improvement in addition performance between pre‐ and post‐tests.

A delayed post‐test conducted 6 weeks after the end of the intervention revealed that the positive effect of the finger training program was robust. Indeed, in non‐finger users who complete all the 3 tests (i.e., pre‐, immediate, and delayed post‐tests), the percentages of finger use and correctly solved additions at delayed post‐test remained higher (75.1%) than at pre‐test (36.2%). In fact, in this population of children, there was no significant decline in performance between immediate pre‐test and delayed post‐test.

While nearly three‐quarters of children in our sample responded positively to the finger training program, it remains that one‐quarter did not. Further investigation is needed to understand why these children did not implement the finger counting strategy taught. Still, some considerations on this point can already be formulated based on the present results. Indeed, it is worth noting that children who did not adopt the finger counting strategy presented a higher percentage of success at pre‐test compared to children who did adopt the strategy (i.e., 51.4% vs. 32.5%). Upon closer examination at the individual level, it was found that 41% of children in this group (9 out of 22) already used an efficient mental strategy at pre‐test (i.e., achieving a minimum of 80% of correctly solved additions). Reassuringly, none of these highly successful children with a mental strategy at the pre‐test switched to using the taught finger counting strategy at the immediate post‐test. It remains that 15% of children in our sample (13 out of 86) were not responsive to our intervention, whereas they could have potentially benefited from it. Indeed, these children displayed 21.5% of additions correctly solved at pre‐test and only 18.5% at immediate post‐test. As a matter of fact, their initial performance was lower than in the whole group (i.e., 37.3%) and it is therefore possible that they did not possess the numerical abilities required to understand and apply the finger counting strategy taught. For example, they may have had limitations in verbal counting, difficulties in number abstraction or in number sense, which hindered their ability to convert symbolic numbers into non‐symbolic quantities on their fingers (Sinclair & Pimm, 2015). Investigating the characteristics of these “non‐responsive” children in future studies would shed light on the generalization of our results to the broader young children population.

Concerning the evolution of the finger counting strategy taught to children, we replicate the observations of Baroody (1987). Indeed, at the immediate post‐test, nearly all children in the experimental group (98%) used the strategy of representing each operand of the problem on each hand. This means that hardly any children shifted to a strategy involving only one hand, which would likely correspond to a COUNT‐ON strategy. Note that children who already used their fingers to solve the problems before our training also massively used the strategy involving both hands (94.3%), confirming that this strategy is the dominant one at the end of kindergarten (Dupont‐Boime & Thevenot, 2018). Reassuringly, children who already counted on their fingers at the beginning of our experiment showed an increase in performance between the pre‐test (84.3%) and the immediate post‐test (92.2%). This improvement was not modulated by the fact that they belonged to the experimental group or not. In other words, our intervention did not negatively affect children's performance when they already counted on their fingers at the beginning of our intervention. This finding also held true when we considered only the very small number of children who already used a strategy involving one hand at pre‐test (*N* = 16).

All in all, our results demonstrate the impressive efficiency of our intervention in improving children's accuracy in an addition task. However, it must be noted that what we taught to children is a procedure for solving additions, and it may not necessarily contribute to a better understanding of the concept of number. In future intervention programs, it will be useful to pre‐ and post‐test important principles associated with the notion of numbers, such as the cardinality or the iteration principles. As noted in our introduction, it is indeed important for children to understand that each of the fingers that they raise represents a unit within a collection and that each of these units represents an “unified all” (Brissiaud, 1992). It is possible that some children in our experiments have not reached such a deep comprehension of the number concept and that, as just stated, what they have learned consists only of a strategy allowing them to reach the correct answer of the addition. Nevertheless, we believe that, as demonstrated in other domains, learning a procedure can enhance the understanding of the concepts underlying this procedure (Greeno et al., 1978, described by Resnick, 1983; Siegler & Stern, 1998). We further think that representing one operand on one hand and one operand on the other to solve simple additions can promote such understanding. This is because this strategy allows the analogical and concrete representations of abstract number symbols through finger configurations that are easily identifiable (e.g., Noël, 2005; Soylu et al., 2019; Thevenot et al., 2014).

Another potential limitation of our approach is that the finger counting strategy that we taught is limited to addition problems with operands up to 5. Nevertheless, echoing our previous point, this strategy likely constitutes a first step toward understanding that a number represents a quantity that can be combined with another quantity, resulting in a whole (e.g., Carpenter & Moser, 1984). However, this approach has been criticized because children could be unprepared and destabilized when confronted with problems such as 7 + 2, where the operand 7 cannot be represented on only one hand. Noticeably, Meljac and Charron (2002) rather promote strategies where the full hand is used as marker to represent quantities (see also Brissiaud, 1992). For instance, in the addition 4 + 3, children would raise 4 fingers on one hand, 1 finger on the same hand, and then 2 other fingers on the other hand, which results in representing the second operand across hands. Although we understand the potential benefit of this strategy, we also believe that its disadvantage is that, at the end of the process, the two problem operands are no longer recognizable on fingers. Therefore, a developmental stage where the analogical representations of each of the operands and the result of their addition are simultaneously visible on fingers might be important, if not necessary, for the comprehension and elaboration of more mature strategies (Thevenot et al., 2001). In our view, the representation of the operands across hands could be taught in a second step, once children master the count‐all strategy on separate hands. As a matter of fact, the results of our present experiment show that, even though the “across hand” strategy is rarely used, it is more often used by children who already count spontaneously on their fingers (i.e., without our intervention) than by children reacting to our intervention (Table 1). These children who already counted on their fingers at the beginning of our experiment may have already moved on to a later stage of finger counting development. This “across hand” strategy could allow them to practice and apply their knowledge concerning number decompositions strategies (i.e., understanding that 4 + 3 is also 5 + 2) (e.g., Cheng, 2012). These reflections and hypotheses will need to be investigated in future experiments and intervention research programs.

## AUTHOR CONTRIBUTIONS

All the authors designed the study; CP, ML, and CT wrote the paper; and CP analyzed the results.

## FUNDING INFORMATION

This work was supported by the Swiss National Foundation for Scientific Research (SNF) under Grant 100014_204271 to Catherine Thevenot.

## Data Availability

The data necessary to reproduce the analyses presented here are publicly accessible at this following URL: https://osf.io/ty53w/?view_only=cff7afaf75124c74925c48d901aed491. Sharing codes or materials do not apply to the present study. The analyses presented here were not preregistered.

## APPENDIX 1 Set of 10 additions presented to children at pre‐, post‐, and delayed post‐tests in Experiment 1 and Experiment 2. In Experiment 3, the 1 + *N* problems were excluded.

**Table:**

1 + 2
1 + 3
1 + 4
1 + 5
2 + 3
2 + 4
2 + 5
3 + 4
3 + 5
4 + 5

## APPENDIX 2 Table used by teachers to report the observations made during the tests in Experiment 1.

**Table:**

ID	Tests	Addition	Child's responses	How many hands the child used to solve the addition? (0/1/2)	How many fingers the child raised up on each hand used?		Verbal protocol
					Left hand	Right hand	
	Pre‐test	1 + 2					
	Pre‐test	1 + 3					
	Pre‐test	1 + 4					
	Pre‐test	1 + 5					
	Pre‐test	2 + 3					
	Pre‐test	2 + 4					
	Pre‐test	2 + 5					
	Pre‐test	3 + 4					
	Pre‐test	3 + 5					
	Pre‐test	4 + 5					
	Immediate post‐test	1 + 2					
	Immediate post‐test	1 + 3					
	Immediate post‐test	1 + 4					
	Immediate post‐test	1 + 5					
	Immediate post‐test	2 + 3					
	Immediate post‐test	2 + 4					
	Immediate post‐test	2 + 5					
	Immediate post‐test	3 + 4					
	Immediate post‐test	3 + 5					
	Immediate post‐test	4 + 5					
	Delayed post‐test	1 + 2					
	Delayed post‐test	1 + 3					
	Delayed post‐test	1 + 4					
	Delayed post‐test	1 + 5					
	Delayed post‐test	2 + 3					
	Delayed post‐test	2 + 4					
	Delayed post‐test	2 + 5					
	Delayed post‐test	3 + 4					
	Delayed post‐test	3 + 5					
	Delayed post‐test	4 + 5					

## APPENDIX 3 Set of 15 additions presented to children during the training sessions in Experiment 1 and Experiment 2.

**Table:**

Session	Additions used during the training sessions	
	Additions used during the demonstration step and during the application	Additions solved with children (presented in a random order)
1	4 + 3	1 + 1 4 + 1 5 + 2 3 + 1
2	2 + 2	3 + 3 4 + 2 5 + 3 5 + 1
3	2 + 1	4 + 4 5 + 5 5 + 4 3 + 2
4	5 + 3	1 + 1 2 + 2 4 + 1 3 + 1
5	4 + 2	3 + 3 4 + 4 5 + 2 2 + 1
6	5 + 5	4 + 3 5 + 1 5 + 4 3 + 2
